# Investigating the Tau proteoform landscape of human brain using a single‐molecule analysis platform

**DOI:** 10.1002/alz70855_101345

**Published:** 2025-12-23

**Authors:** Andreas Huhmer, Zhengjian Zhang, Vivek Budamagunta, James Joly, Sanjib K Guha, Rosemary Wang, Steven Tan, Kara Juneau, Parag Mallick

**Affiliations:** ^1^ Nautilus Biotechnology, San Carlos, CA, USA

## Abstract

**Background:**

The microtubule‐associated protein Tau (MAPT) is implicated in various progressive neurodegenerative diseases, including Alzheimer's Disease (AD). With six primary splicing isoforms and over 70 different post‐translational modifications (PTMs) identified on Tau, its proteoform diversity is extensive. However, a significant knowledge gap remains regarding the prevalence of specific proteoforms and their role in the progression of neurodegenerative diseases.

**Method:**

We employed a novel single‐molecule assay utilizing the Nautilus proteome analysis platform to investigate the Tau proteoform landscape in human brain samples. This platform immobilizes individual protein molecules on a hyper‐dense flow cell, which are then probed iteratively with splice‐variant specific or PTM‐specific antibodies. Each Tau molecule's proteoform is estimated based on the pattern of antibody binding, with the overall abundance of each proteoform quantified by advanced data‐processing software that corrects for potential binding errors and off‐target interactions.

**Result:**

We analyzed brain samples from seven individuals, five diagnosed with Alzheimer's and two controls, assessing the diversity and abundance of Tau proteoforms. Our findings reveal a complex proteoform landscape with distinct differences in the maturity and phosphorylation patterns of Tau isoforms between affected individuals and controls. Notably, hyperphosphorylation was prevalent in samples from Alzheimer's patients, with clear differentiation between proteoforms with minimal phosphorylation and those with extensive modifications.

**Conclusions:**

The single molecule platform has proven effective in quantifying the molecular heterogeneity of Tau proteoforms in human brain tissue. This analysis begins to enhance our understanding of Tau's role in Alzheimer's disease, highlighting its potential for developing more sensitive diagnostic approaches based on proteoform heterogeneity. This study sets the stage for future research on the impact of specific Tau proteoforms on neurodegenerative disease progression and their utility as biomarkers for AD.

